# Identification of proteins related to the stress response in *Enterococcus faecalis *V583 caused by bovine bile

**DOI:** 10.1186/1477-5956-8-37

**Published:** 2010-06-25

**Authors:** Liv Anette Bøhle, Ellen M Færgestad, Eva Veiseth-Kent, Hilde Steinmoen, Ingolf F Nes, Vincent GH Eijsink, Geir Mathiesen

**Affiliations:** 1 Department of Chemistry, Biotechnology and Food Science, Norwegian University of Life Sciences, Chr. M. Falsensvei 1, N-1432 Ås, Norway; 2 Nofima Mat AS, Norwegian Institute of Food, Fisheries and Aquaculture Research, N-1430 Ås, Norway

## Abstract

**Background:**

*Enterococcus faecalis *is an opportunistic pathogen and one of the most important causes of hospital infections. Bile acids are a major stress factor bacteria have to cope with in order to colonize and survive in the gastro-intestinal tract. The aim of this study was to investigate the effects of bile acids on the intracellular proteome of *E. faecalis *V583.

**Results:**

The proteomes of cells challenged with 1% bile were analyzed after 20 - 120 minutes exposure, using 2D gel electrophoresis and mass spectrometry. Among the approximately 500 observed proteins, 53 unique proteins were found to be regulated in response to bile and were identified with mass spectrometry. The identified proteins belonged to nine different functional classes, including fatty acid- and phospholipid-biosynthesis, energy metabolism, and transport and binding. Proteins involved in fatty acid and phospholipid biosynthesis pathways were clearly overrepresented among the identified proteins and all were down-regulated upon exposure to bile. The proteome data correlated reasonably well with data from previous transcriptome experiments done under the same conditions, but several differences were observed.

**Conclusion:**

The results provide an overview of potentially important proteins that *E. faecalis *V583 needs to regulate in order to survive and adapt to a bile-rich environment, among which are several proteins involved in fatty acid and phospholipid biosynthesis pathways. In addition, this study reveals several hypothetical proteins, which are both abundant and clearly regulated and thus stand out as targets for future studies on bile stress.

## Background

*Enterococcus faecalis *is a wide-spread Gram-positive lactic acid bacterium, and is a natural inhabitant of the gastrointestinal tract (GIT) of humans and animals. The bacterium is also commonly found in soil, sewage, water and food. *E. faecalis *V583 is an opportunistic pathogen that can cause diseases like urinary tract infections, bacteremia, and infective endocarditis in immunocompromised patients. These infections may be problematic because *E. faecalis *strains tend to be resistant toward many antibiotics, including vancomycin [1,2]. Vancomycin-resistant enterococci were first found among clinical isolates in the late 1980s, and antibiotic resistance has increased since. Infections by enterococci have become a major problem in the hospital environments and enterococci are now ranked among the most prevalent nosocomial pathogens [3,4].

*E. faecalis *is able to grow and colonize many hostile environments including the GIT, and is considered as an interesting model for studying bacterial stress responses [5]. It is important to understand such responses in enterococci because the ability to survive in a wide range of environments obviously contributes to enterococcal prevalence in e.g. hospital environments. In order to survive in the human GIT, bacteria must overcome several adverse environmental stresses such as low pH, low oxygen levels, nutrient limitations, elevated osmolarity and the deleterious actions of bile. The liver daily secretes about one liter of bile, which consists mainly of bile acids, cholesterol, phospholipids, and the pigment biliverdin. In the human GIT, bile acts as a biological detergent, emulsifying and solubilising fats [6]. Exposure to bile may lead to changes in the fatty acid- and phospholipid-composition of bacterial membranes and to distortion of the cell surface [7,8]. It has also been shown that bile can induce secondary structure formation in RNA, induce DNA damage and activate enzymes involved in DNA repair [6].

Various studies indicate that bacteria thriving in the GIT, such as lactic acid bacteria and bifidobacteria, have evolved mechanisms to protect themselves from the noxious effects of bile. Genome-wide and gene-by-gene studies have shown that Gram-positive bacteria such as listeria, lactobacilli and bifidobacteria carry genes coding for transporters able to extrude bile salts, the expression of which is regulated by bile salts [9-11]. Additional genes whose expression is regulated by bile include genes involved in more general stress responses and genes involved in carbohydrate metabolism and fatty acid biosynthesis [12-15].

While genome and transcriptome analyses have provided interesting clues as to how enterococci manage bile stress, so far, proteome information is limited [16-18]. Since transcriptome and proteome data do not necessarily correlate, knowledge of proteome responses is a prerequisite for obtaining a more complete picture of the bile salt response. Therefore, we have analyzed how the intracellular proteome of *E. faecalis *V583 responds to bovine bile in a time course experiment, assessing both immediate responses and longer term effects. We used two-dimensional- (2D) gel electrophoresis combined with mass spectrometry-based protein identification to identify the bacterial proteins whose abundances change during growth in presence of 1% bovine bile. The experiments were conducted using conditions identical to those used in a previous transcriptome study on bile stress [15] permitting comparison of proteome and transcriptome data.

## Materials and methods

### Bacterial strain and sample collection

*E. faecalis *V583 [19] was grown in brain heart infusion (BHI) medium (Oxoid Ltd., Hampshire, England) aerobically, over-night, with shaking, 300 rpm, at 37°C. The over-night cultures (50 ml) were diluted 50-fold in 100 ml BHI (37°C) and grown further to an OD _600 _of ~0.2, after which the cells were collected by centrifugation (9800 × g, 10 min, at room-temperature). After resuspending the cells in 50 ml of fresh prewarmed BHI (37°C) another 50 ml of prewarmed BHI-medium with or without bovine bile (Sigma-Aldrich Inc, St. Louis, MO) was added. Thus, the final volume was 100 ml for each condition and the final concentration for the samples with bovine bile was 1% (w/v). The cultures were incubated with shaking, 300 rpm, at 37°C, until harvesting. After incubation for 20, 60 or 120 minutes cells were harvested from the cultures by centrifugation (9800 × g, 10 min, 4°C). The cell pellets were washed three times with ice-cold 0.9% (w/v) NaCl, and subsequently resuspended in a lysis- and rehydration-solution containing 8 M urea, 2 M thiourea, 50 mM DTT and 2% CHAPS. The cells were disrupted with glass-beads (106 micron, Sigma-Aldrich) using a FastPrep-24 instrument (MP Biomedicals, Solon, OH) (speed 6, three treatments of 45 seconds each, with 60 second pauses in between, at 4°C). The resulting cell-free extracts were stored at -20°C until the 2D analyses were performed (see below). The protein concentration of the cell-free extracts was measured using the Bradford Microassay (Bio-Rad Laboratories, Inc, Hercules, CA).

To check the effect of bile on the growth rate of *E. faecalis *V583, the bacteria were grown in liquid BHI with or without 1% bile, at 300 rpm, 37°C. The growth was followed by measuring the optical density (OD _600_) of the cultures every 20 minutes in the first 3 hours, and then every 30 minutes.

### Two-dimensional gel electrophoresis and analysis

Isoelectric focusing (IEF) was performed using 24 cm IPG-strips (Bio-Rad) covering the pH-area 4-7. The cell-free protein sample was diluted in a lysis-and rehydration-solution together with ampholytes in a total volume of 450 μl, containing 75 μg proteins. This protein-sample was loaded on the strips, and rehydration was performed for 16 hours at 50 V using a Protean IsoElectric Focusing Cell II unit (Bio-Rad). Then, isoelectric focusing was carried out using the following voltage program: a linear ramp to 250 V over 30 minutes, followed by a rapid ramp to 500 V over 1 hour, a rapid ramp to 1000 V over 1 hour, and then a rapid ramp to 10000 V, where the voltage is fixed until the total reached 70000 Vh.

To prepare for electrophoresis in the second dimension, the IPG-strips were first equilibrated in a buffer containing 6 M urea, 50 mM Tris-HCl pH 8.8, 30% glycerol, 2% SDS and 1% dithiothreitol (DTT) for 15 minutes to reduce sulfhydryl groups, and then in a buffer containing 6 M urea, 50 mM Tris-HCl pH 8.8, 30% glycerol, 2% SDS and 5% iodoacetamide for an additional 15 minutes to alkylate the reduced sulfhydryl groups. Subsequently, the strips were loaded onto a 12.5% SDS-gel for the SDS-PAGE step. The electrophoresis was run at 5 mAmp/gel for 3 hours and then at 15 mAmp/gel for 12 hours. Normally, 12 gels were run simultaneously, using an EttanDALT Electrophoresis system (Amersham Biosciences, USA). The gels were silver stained according to a previously described method [20].

For each growth condition/time point (six combinations in total), five biological replicates were produced. These 30 samples were run twice through the 2D-electrophoresis, yielding a total of 60 gels. Spot detection and gel alignment were performed using Delta2D software (DECODON, Greifswald, Germany). In this method all pixels are compiled into a fused image that is used for setting common spot boundaries for all gel samples, whereas the raw data for the spot intensities are obtained from each individual image [21]. All proteins were visually checked and only spots that did not appear as streaking were included (this procedure was repeated after pre-selection of regulated proteins; see Results section). In the further analyses only spots showing a fold change of at least 1.9 (with bile versus without bile) at at least one time point were included. This somewhat arbitrary threshold value is comparable to the threshold factors used in other studies [22,23]. Lower threshold factors were not considered in order to maximize the chance that the discovered changes in protein levels are not only statistically but also biologically significant. Based on this criterion, 115 spots were selected and subjected for statistical analysis. Analysis of variance was performed where the p-values were adjusted for multiple comparisons by False Discovery Rate (FDR) using rotation test [24,25] with a significance level of p < 0.05. Upon this procedure, the number of spots was reduced to 91.

### Protein identification

Spots representing differently expressed proteins were excised from the gel and the gel pieces were washed 2 × 15 minutes with a 1:1 mixture of acetonitrile (ACN) and 50 mM ammonium bicarbonate. The gel-pieces were dried in a speed-vac and the dried gel-pieces were reswollen with 0.03 μg/μl trypsin at 4°C for 30 minutes (approximately 5 μl on average). Subsequently, 25 μl 50 mM ammonium bicarbonate was added, and the digestion of proteins was performed by incubating overnight at 37°C with shaking at 300 rpm. The peptides were extracted from the gel-pieces by incubating the pieces in 1% trifluoroacetic acid (TFA), 0.1% (v/v) TFA in 50% (v/v) ACN, and 100% ACN, in three consecutive steps. The liquid phases were pooled and the extracted peptides were dried in a speed-vac, rehydrated in 0.1% (v/v) TFA, and sonicated prior to desalting using C18 STAGE tips [26]. After eluting the peptides with 1 μl 65% (v/v) ACN, 0.5 μl of the eluate was mixed with 0.5 μl matrix solution (α-cyano-4-hydroxycinamic acid), and spotted onto a MALDI target plate (Bruker Daltonics, Billerica, MA).

Peptide mass fingerprints (PMF) and MS/MS fragmentation spectra were determined using an UltraflexMALDI-TOF/TOF (Bruker Daltonics) instrument. Protein identification was carried out with Mascot (Matrix Science Inc., Boston, MA), limiting the search to bacteria belonging to the "Other Firmicutes" in the NCBI database. The searches were limited to only consider tryptic fragments, with carbamidomethylation of cysteine (fixed modification) and possible oxidation of methionine. The error tolerance was set to 50 ppm, and the number of allowed missed cleavage sites was set to 2.

Some proteins giving weak spots could not be identified by MALDI-TOF/TOF. These proteins were analyzed by Nano LC coupled to ESI-MS/MS, using an LC-LTQ Orbitrap-MS at The Biotechnology Centre of Oslo, Norway.

## Results

**Figure 1:**
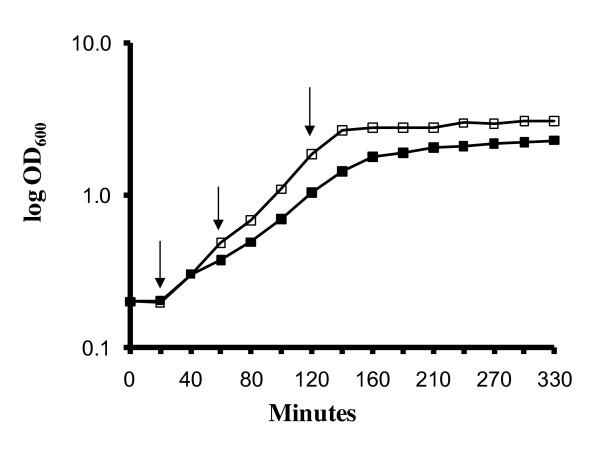
**Growth-curve of *E. faecalis *V583 in liquid BHI medium with (black square) or without 1% bovine bile (open square)**. Growth was measured as absorbance at 600 nm. Each time point represents the mean value of three biological replicates. The arrows indicate the time points (20, 60 and 120 minutes) where the cells were harvested for proteome analysis.

The effect of bovine bile on the growth rate of *E. faecalis *V583 was tested by comparing growth in the absence and presence of 1% (w/v) bovine bile in liquid BHI medium (Fig. 1). The growth curves showed that the presence of bile reduces both the growth rate and the maximum cell density, but also showed that the levels of bile used in this study are clearly sub-lethal, permitting accumulation of sufficient cells in the samples with bile. Cells were harvested at 20, 60 and 120 minutes after the addition of bile.

Additional file 1 shows six representative gels from the 2D-gel electrophoresis experiments, one gel for each time point at each condition (presence or absence of bile). Visual inspection of the about 500 distinguishable spots on the gels showed some clear differences between the bile and non-bile samples. The data-set used for image analysis and identification of differentially expressed proteins was obtained from 60 gels. These gels represented five biological replicates for each growth condition/time point (30 samples in total), which were run twice. Statistical validation was performed based on the pixel values for each protein spot. In total 91 spots met the two criteria of (1) showing significant changes according to the FDR adjusted p-value (<0.05) and (2) showing an increase or decrease of at least 1.9-fold in response to bile at at least one of the three time points. The 91 spots were visually controlled in more detail and proteins that appeared as streaking were removed from the analysis (32 spots; these are spots for which the image analysis is prone to errors). The resulting 59 spots were subjected to protein identification by mass spectroscopy. This analysis identified 55 proteins whose abundances were considered to change upon bile stress while four proteins could not be identified. Two proteins were identified twice: spot 16 and 19 were identified as formate acetyltransferase (EF1613), and spot 49 and 51 were identified as ribose-phosphate pyrophosphokinase (EF3163). In both cases, the two protein spots belonging to the same protein represented different masses and pI. Such differences may be explained by one of the spots being a proteolytic fragment or carrying another type of post-translational modification. Within each pair, the spots showed similar trends in terms of the change in protein abundance.

**Figure 2:**
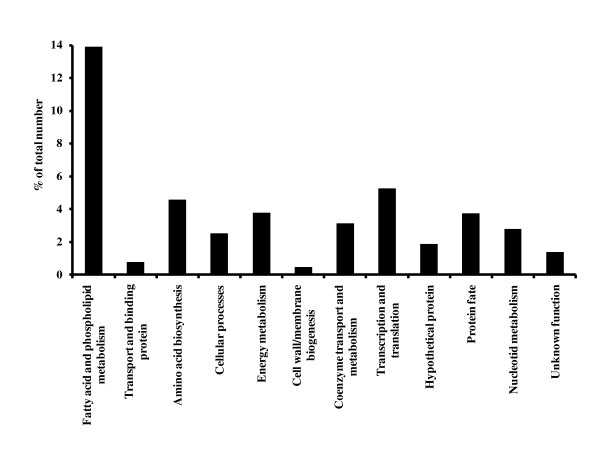
**Relative abundance of identified regulated proteins in *E. faecalis *V583**. Proteins (53 in total) are grouped according to their functional class as defined by JVCI http://cmr.jcvi.org/tigr-scripts/CMR/CmrHomePage.cgi. The values for each class represent the fraction (in %) of the total expected proteome in that class that was identified in the present study.

**Figure 3:**
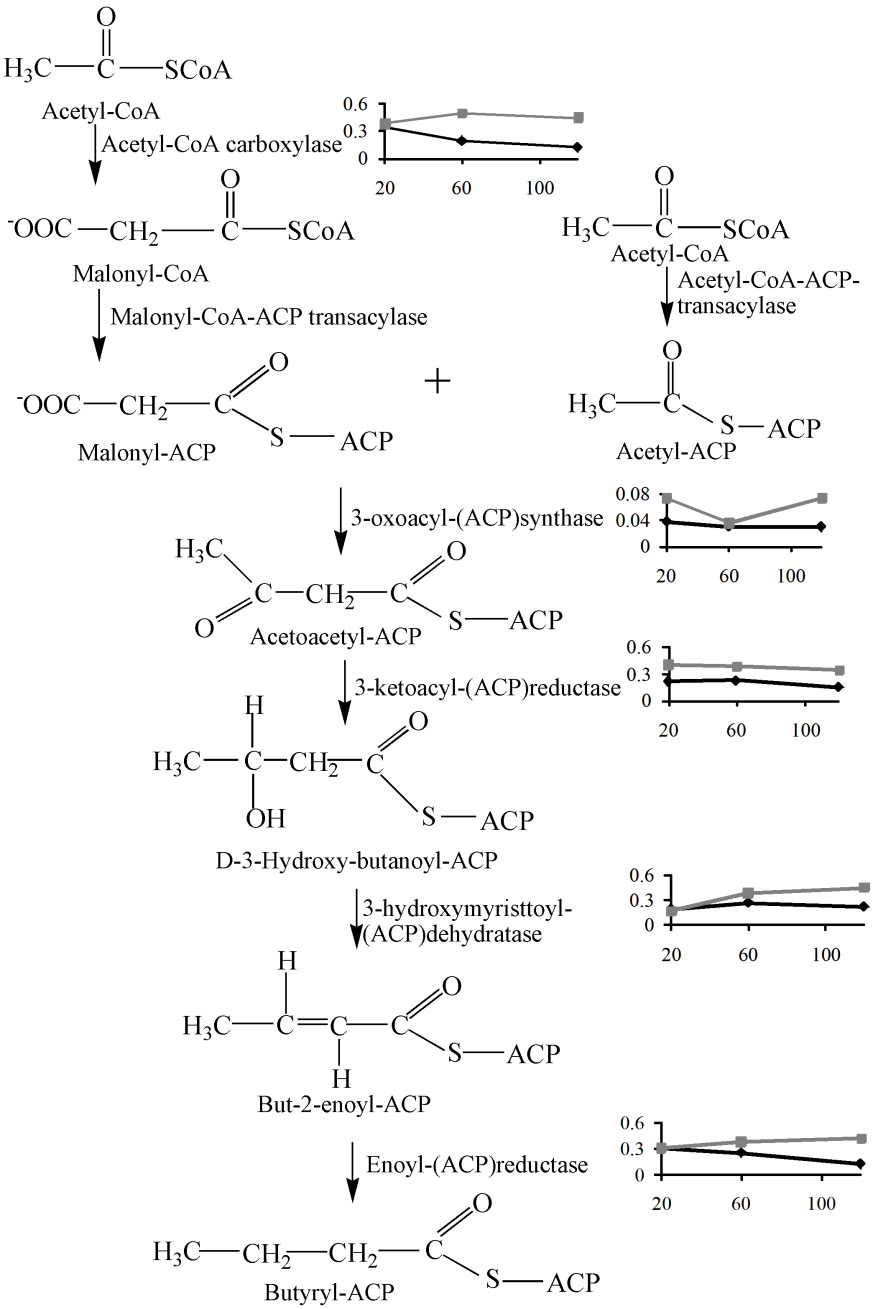
**Overview of fatty acid biosynthesis http://www.genome.jp/kegg/pathway/map/map00061.html and regulation of key enzymes**. The starting reactant is acetyl-CoA. The principal reaction products of the fatty acid biosynthesis are straight-chain C16 and C18 fatty acids, which can be used in the synthesis and the repair of damaged phospholipid membranes. The resulting fatty acids are therefore important constituents of the cell membrane. The graphs to the right show the development of protein abundance during the time course experiment. The grey line corresponds to protein produced in bacteria grown in media without bovine bile, while the black line corresponds to bacteria grown in media containing 1% bovine bile. The x-axis indicates the time (minutes) and the y-axis the spot intensity (normalized raw spot volume) of the protein as a mean value of the parallels. The lines are drawn for illustration purposes only, connecting the three time points that were analyzed (20, 60 and 120 minutes).

Of the 53 identified unique proteins, 17 were up-regulated, 32 were down-regulated, and four were both up- and down-regulated during the time course experiment (Table 1). The number of regulated proteins increased during the time course experiment, from eight after 20 minutes, to 21 after 60 minutes, to 38 after 120 minutes (Table 1). Only 14 proteins showed significantly altered abundances at more than one time point, indicating that the observed up- and down-regulations are relatively transient. Among the 53 unique proteins, 14 are annotated in the J. Craig Venter Institute (JCVI) Comprehensive microbial resource as hypothetical proteins or proteins with unknown functions (Table 1). The other 39 proteins belong to nine different functional classes (Table 1). Proteins involved in energy metabolism and in the transcription-translation process were most abundant, with eight and nine identified proteins in each functional class, but their relative abundance was comparable with that of proteins in most of the other functional classes (Fig. 2). These two most numerous groups did not show clear overall trends with respect to up- or down-regulation. Proteins involved in fatty acid and phospholipid metabolism were clearly overrepresented among the identified proteins (Fig. 2) and all of these were down-regulated by the presence of 1% bovine bile (Fig. 3). The rest of the identified proteins are predicted to be involved in processes related to protein fate, transport and binding, nucleotide metabolism, coenzyme transport and metabolism, amino acid biosynthesis, and cell wall/membrane biogenesis, as well as in "other cellular processes" related to bacterial adaptation to atypical conditions.

**Table 1:** Identified proteins of E. faecalis V583 whose abundance is affected by the presence of 1% bovine bile—the three columns to the right show the results for cells harvested at three different time points (20, 60 and 120 minutes)

Spot no.	**Functional class**^***a***^	**Putative function**^***a***^	ORF	Mass (kDa)	**pI**^***b***^	**Coverage**^***c***^	No. of peptides matched	**Fold ratio**^***d ***^ T(20) T(60) T(120)		
**1**^***e***^	Fatty acid and phospholipid metabolsim	Enoyl-(acyl carrier protein) reductase	EF0282	26.9	5.29	42	14	1.01	0.67	**0.3**
**2**^***e***^		Acetyl_coA carboxylase, biotin carboxyl carrier protein	EF2879	17.6	4.23	40	8	0.92	**0.39**	**0.28**
**3**^***e***^		(3R)-hydroxymyristoyl-(acyl-carrier-protein) dehydratase	EF0284	16.4	5.73	22	5	1.09	0.71	**0.49**
**4**^***e***^		3-oxoacyl-(acyl-carrier-protein) synthase II	EF0283	43.5	5.11	40	12	**0.51**	0.87	**0.41**
**5**^***e***^		3-ketoacyl-(acyl-carrier-protein) reductase	EF2881	26.1	5.92	35	10	1.70	**0.37**	0.55
**6**	Transport and binding protein	Phosphocarrier protein HPr	EF0709	9.3	4.92	13	11	0.99	0.70	**0.52**
**7**^***e***^		PTS system, mannose-specific IIAB components	EF0020	35.5	5.11	50	22	1.62	**3.06**	0.90
**8**		peptide ABC transporter, ATP-binding protein	EF0912	35.9	5.97	20	7	1.10	1,00	**0.46**
**9**^***e***^	Amino acid biosynthesis	Ornithine carbamoyltransferase	EF0105	38.1	5.02	18	5	1.03	0.64	**3.48**
**10**^***e***^		Decarboxylase, putative	EF0634	71.96	5.14	47	29	**0.52**	1.21	1.86
**11**	Other cellular processes	Glutathione reductase	EF3270	49.6	5.23	21	10	**2.42**	0.96	0.87
**12**		Dps family protein	EF3233	17.9	4.56	46	6	**0.51**	0.81	1.23
**13**		General stress protein, putative	EF1744	20.5	4.61	31	6	0.88	0.66	**0.52**
**14**		Cell division protein DivIVA	EF1002	26.6	4.53	62	16	0.61	1.21	**3.5**
**15**^***e***^	Energy metabolism	Deoxyribose-phosphate aldolase	EF0174	23.3	4.65	48	10	1.39	1.28	**0.47**
**16**^***f***^		Formate acetyltransferase	EF1613	84.5	5.31	11	8	1.00	0.95	**2.87**
**17**^***e***^		Fumarate reductase flavoprotein subunit	EF2556	53.8	5.26	10	6	0.86	1.57	**2.26**
**18**		Phosphoglycerate mutase 1	EF0195	26.0	5.09	41	11	0.87	1.13	**0.36**
**19**^***f***^		Formate acetyltransferase	EF1613	84.7	5.31	14	11	0.94	1.21	**2.09**
**20**^***e***^		V-type ATP synthase subunit B	EF1499	51.3	5.03	18	8	0.59	**2.02**	1.29
**21**		Pyruvate kinase	EF1046	62.6	4.99	41	25	**2.99**	**3.61**	1.09
**22**		Enolase	EF1961	46.5	4.56	54	22	1.46	0.98	**0.45**
**23**		Thioredoxin	EF1405	11.7	4.35	66	9	1.49	**0.40**	0.60
**24**^***e***^	Cell wall/membrane biogenesis	D-fructose-6-phosphate amidotransferase	EF2151	65.7	4.93	24	12	**1.93**	1.13	**2.12**
**25**	Coenzyme transport and metabolism	Naphthoate synthase	EF0445	30.0	5.24	39	10	0.89	1.46	**0.43**
**26**^***e***^		2-dehydropantoate 2-reductase	EF2445	34.7	5.08	3	1 (39)*^g^*	1.32	1.40	**0.46**
**27**^***e***^	Transcription and translation	Transcriptional regulator, AraC family	EF0432	34.6	6.76	25	7	0.91	**2.64**	**0.18**
**28**		Cold-shock domain-contain protein	EF2925	7.3	4.35	60	5	0.97	**0.33**	**0.37**
**29**		Peptide deformylase	EF3066	21.0	5.08	48	6	1.15	0.77	**0.5**
**30**		30S ribosomal protein S2	EF2398	29.5	5	24	8	**2.82**	1.87	0.81
**31**^***e***^		Ribosomal protein L31	EF1171	10.1	5.57	95	10	1.65	**0.36**	**0.29**
**32**		Elongation factor G	EF0200	76.6	4.8	23	19	0.93	0.87	**1.97**
**33**		30S ribosomal protein S3	EF0212	24.4	9.8	12	3	0.86	**2.19**	0.68
**34**		Phenylalanyl-tRNA synthetase subunit beta	EF1116	88.8	4.76	12	11	0.87	0.97	**2.18**
**35**		Methionyl-tRNA formyltransferase	EF3123	34.3	6.06	3	1 (48)*^g^*	0.96	1.29	**0.41**
**36**	Hypothetical protein	Hypothetical protein EF1967	EF1967	20.7	5.98	20	5	**0.51**	0.86	**0.25**
**37**		Hypothetical protein EF2909	EF2909	12.1	4.33	52	7	0.99	**0.47**	0.69
**38**		Hypothetical protein EF2763	EF2763	12.1	4.7	43	5	1.25	**0.43**	0.66
**39**		Hypothetical protein EF2888	EF2888	8.9	4.54	42	6	1.07	**0.32**	**0.43**
**40**^***e***^		Hypothetical protein EF3184	EF3184	26.4	4.86	28	7	0.80	**0.31**	**0.38**
**41**^***e***^		Hypothetical protein EF3186	EF3186	25.7	4.91	28	8	1.44	1.80	**0.44**
**42**		Hypothetical protein EF0123	EF0123	85.5	7.23	4	3	1.89	**2.11**	**0.43**
**43**		Hypothetical protein EF0352	EF0352	29.3	6.91	4	1 (61)^g^	1.44	**2.18**	**0.48**
**44**		Hypothetical protein EF2174	EF2174	99.6	8.68	36	33	1.54	1.41	**0.32**
**45**		Hypothetical protein EF 2104	EF2104	43.6	4.88	18	6	0.82	**2.36**	1.17
**46**	Protein fate	Glutamyl-aminopeptidase	EF3037	39.4	5.68	22	8	0.87	**1.90**	1.01
**47**^***e***^		DnaK protein	EF1308	65.5	4.59	56	31	1.11	1.20	**2.21**
**48**^***e***^		Heat shock protein GrpE	EF1307	20.1	4.5	26	8	1.10	**0.30**	**0.30**
**49**^***f***^	Nucleotid metabolism	Ribose-phosphate pyrophosphokinase	EF3163	35.4	6.16	22	9	1.21	**2.5**	**0.47**
**50**		Inositol-5-monophosphate dehydrogenase	EF3293	52.8	5.7	50	84	1.24	**2.0**	1.05
**51**^***f***^		Ribose-phosphate pyrophosphokinase	EF3163	35.5	6.16	15	7	0.95	1.21	**0.38**
**52**	Unknown function	DNAbinding response regulator VicR	EF1193	26.9	5.17	15	6	0.87	1.20	**0.51**
**53**		Glyoxalase family protein	EF2591	31.7	4.85	50	15	1.04	0.99	**0.30**
**54**		PhnA protein	EF1374	12.4	5.01	66	6	0.9	0.55	**0.47**
**55**		Oxidoreductase, aldo/keto reductase family	EF1138	31.0	5.28	18	5	0.97	1.22	**0.44**

^a ^The putative function is based on the JCVI Comprehensive Microbial Resource database http://cmr.jcvi.org/tigr-scripts/CMR/CmrHomePage.cgi.

^b^The pI values are theoretical values calculated from the protein sequences

^c ^The coverage shows the percentage of the protein that is covered by the identified peptides.

^d ^Proteins with values over 1 are up-regulated in response to bile, proteins with values below 1 are down-regulated. Values representing a change larger than 1.9-fold are printed in bold face.

^e ^Proteins that also were shown to be regulated in a transcriptome study of bile responses in E. faecalis V583 [15]; see text for details.

^f ^Note that proteins EF3163 (spots 49 & 51) and EF1613 (spots 16 & 19) were identified twice.

^g^The value in parenthesis shows the probability-based Mowse score for proteins that were identified by only one peptide (in all cases using the LC-LTQ Orbitrap). The Mowse score equals -10*Log (P), where P is the probability that the observed match is a random event; Mowse scores greater than 38 indicate identity or extensive homology (p < 0.05).

**Figure 4:**
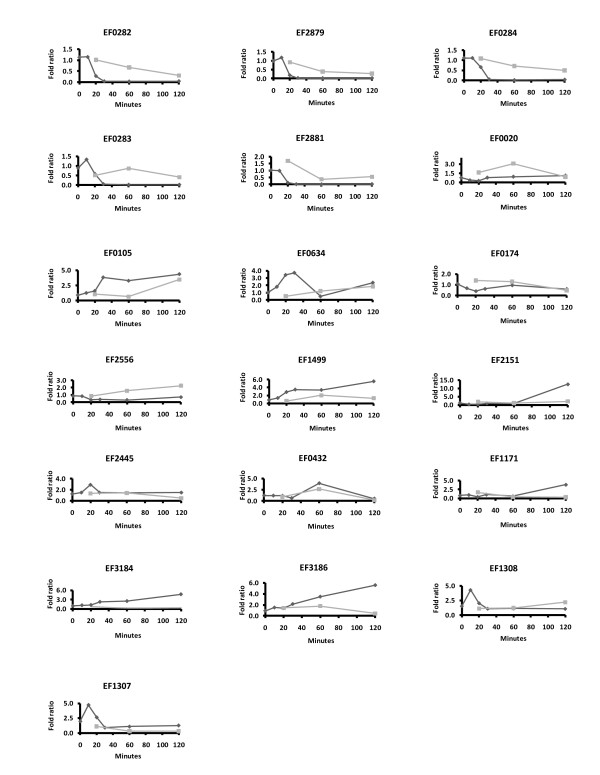
**Expression-profiles of the 19 proteins that were found to be regulated by bile at both the transcriptome **[15]**and the proteome level**. The dark lines show the data from the transcriptome study, while the grey lines show the data from the present proteome study. The y-axis indicates the fold ratio, while the x-axis shows the culturing time in minutes.

The growth conditions used in the present proteome study were similar to those used in a previous transcriptome study with the same bacterium [15]. The expression profiles from the transcriptome study [15] were compared with the data from the present study for the 19 proteins that were found to be significantly regulated in both studies (Fig. 4). While the expression profiles derived from the two methods show the same tendencies for about eleven of these 19 proteins, discrepancies are observed in several cases, either at one time point (e.g. EF0020 & EF1499 at 120 minutes) or in the form of an overall trend (e.g. EF3184 and EF3186 where an increase in transcription does not seem to lead to an increase in protein).

## Discussion

In the present study we used 2D gel electrophoresis to identify proteins in *E. faecalis *V583 whose levels are regulated in response to bile. The cells were analysed at three different time points. By using this set up both the initial and more long-term effects of bile could be observed.

The gels showed approximately 500 spots, which is 20% of the intracellular proteome of *E. faecalis *V583. Use of visual inspection, a threshold fold ratio of 1.9, and rigorous statistical analysis led to the identification of 53 unique proteins that are regulated in response to bile stress, representing about 10% of the proteins on the gels. This number of regulated proteins is higher than what has been obtained in similar proteomic studies in other Gram-positive bacteria. Previous studies have identified 28 proteins in *Lactobacillus reuteri *[27], 24 proteins in *Propionibacterium freudenreichii *[22], 45 proteins in *Bifidobacterium animalis *[28], and 34 proteins in *Bifidobacterium longum *[23]. The high number obtained in the present study may partly be due to the fact that we have studied the proteome in a time-course experiment, which, to the best of our knowledge, has not been done before in studies of bile responses in Gram-positive bacteria.

Bile stress in *Enterococcus faecalis *was among the first bacterial stress responses to be studied using the 2D electrophoresis approach. In an early study aimed at identifying stress-induced proteins, 45 protein spots were found to be up-regulated upon exposure to bile for 30 minutes [16]. Only two proteins, DnaK and GroEL, were identified and it is therefore difficult to compare this work with the present study (note that DnaK was also found to be upregulated in our study; see below). In a later study by Giard et al. [18], peptide sequences for four additional bile-induced proteins were presented which correspond to EF0453, a protein involved in hydrogen peroxide resistance, and to three hypothetical proteins (EF0770, EF1560, EF2798). None of these four proteins were found to be significantly regulated in the present study. This difference may be due to the fact that the bile salt concentrations used in these previous studies were lower (0.08 or 0.3%). Furthermore, these studies used another *E. faecalis *strain which is less resistant to bile than the V583 strain.

The 2D-PAGE experiments provide information on changes in protein levels only and do not provide direct information on the underlying gene expression levels (mRNA production levels). The previous transcriptional study [15] on the same bacterium and under the same conditions, revealed 308 genes whose expression was affected by bile during the time course experiment, representing about 10% of the genes in the genome. The fraction of regulated genes in each functional class varied between 5.6% and 16.7% of the total number of genes per class, with the exception of genes involved in the fatty acid- and phospholipid metabolism, of which 41.7% were found to be regulated [15]. Compared to the transcriptome study, the present proteome study revealed fewer regulated genes/proteins; we identified about 2% of the total proteome and the representation of each functional class varied between 0.5% and 5.3% (Fig. 2). Again, proteins involved in fatty acid- and phospholipid metabolism formed the exception, since 14.0% of these were found to be regulated. The lower number of genes/proteins found in the proteome study is not only due to sensitivity and dynamic range issues but also to the fact that insoluble proteins and the major part of proteins that are secreted out of the cell are not expected to be found in the cytoplasmic fraction. The transcriptome and proteome data correlate reasonably well for 11 of the 19 genes/proteins that were identified with both methods (Fig. 4). Not unexpectedly, in several cases changes in protein levels lag behind changes in RNA levels. Several proteins show clear discrepancies between the proteome and the transcriptome data and in almost all of these cases an upregulation of the mRNA is not accompanied by higher protein levels (EF1499, EF2151, EF1171, EF0020, EF3184, EF3186). One may speculate that other regulatory mechanisms come into play, such as translational regulation or specific protein degradation. Two of these genes (EF3184, EF3186) are part of an operon consisting of five genes, of which four encode proteins with putative N-terminal signal-peptides. None of these proteins show homology with other proteins with known function. Clearly, the fact that these proteins are secreted may lead to discrepancies between the transcriptome data and data for the intracellular proteome.

Proteins related to general bacterial stress responses primarily occur in the functional classes "Protein fate" and "Other cellular processes". We identified two general stress proteins, the chaperone DnaK (EF1308) and a heat-shock protein GrpE (EF1307). It has previously been shown that a number of stress factors, including bile, have an effect of the expression of DnaK and/or GrpE in *Enterococcus *and other Gram-positive bacteria [16,18,29,30]. Three of the four identified proteins in "Other cellular processes" are related to stress; glutathione reductase (EF3270), Dps (DNA protecting protein under starved conditions; EF3233), and a putative general stress protein of unknown function (EF1744). Whereas EF3233 and EF1744 both are down-regulated, the glutathione reductase, a cellular antioxidant involved in oxidative defence, is significantly up-regulated after 20 minutes, indicating fast response to the deleterious effects of bovine bile. The fourth protein, DivIVA (EF1002) is strongly up-regulated after 120 minutes. DivIVA plays a crucial role in cell division [31]. Recently, it has been shown that DivIVA may also play a role in managing oxidative stress [32]. Other (transcriptome) studies have also shown that expression of general stress proteins is influenced by bile acids [12,13].

The five proteins involved in the fatty acid biosynthesis affected by bile correspond to 14% of the total number of proteins in the functional class of "Fatty acid and phospholipid metabolism", meaning that this class is clearly overrepresented among the regulated proteins (Fig. 2). These five proteins, as well as nine of the ten additional affected genes within this class identified in the previous transcriptome study [15], were all down-regulated upon exposure to bile and the down-regulation increased during the time course experiment (Table 1). All five proteins identified here are involved in fatty acid biosynthesis (Fig. 3), and our data thus clearly show that this process is down-regulated in response to bile. Previous studies have suggested that bile changes the membrane surface as a response to the actions of bile [7,33], but so far, there is limited proteomic data that has supported this notion. Proteomic studies of bile stress in *B. animalis *[28] and *P. freudenreihii *[22] each led to the identification of only one (down-regulated) protein in the "Fatty acid and phospholipid metabolism" class. Interestingly, a recent transcriptome study showed that expression of genes involved in fatty acid and phospholipid biosynthesis was up-regulated in *E. faecalis *V583 grown on blood [34]. This indicates that the down-regulation of fatty acid biosynthesis is a bile-specific response and that the bacteria respond differently when grown in blood.

Eight regulated proteins play a role in energy metabolism, representing 4% of this functional class (Fig. 2). The proteins are involved in different pathways, including glycolysis (EF 0195, EF1046, EF1961), pentose phosphate pathway (EF0174), pyruvate metabolism (EF1613), oxidative phosphorylation (EF2556, EF1499), and the electron transport chain (EF1405). Proteins identified in the first two pathways are members of the carbohydrate metabolism, and most of these proteins were down-regulated in contrast to what has been observed in other studies regarding bile stress [23,27]. Interestingly, a V-type ATPase (EF1499), which couples hydrolysis of ATP to the translocation of protons across bacterial membranes, was up-regulated. Maintenance of the proton motive force is an important factor during bile stress, because it contributes to sustain cellular homeostasis [6]. Pyruvate kinase (EF1046), which converts phosphoenolpuruvate to pyruvate, and formate acetyltransferase (EF1613), which is responsible for the transfer of an acetyl group from acetyl-CoA to formate, yielding CoA and pyruvate, were up-regulated. Pyruvate is a metabolic key molecule that can be used in a number of different reactions to increase the ATP levels. The combined up-regulation of formate acetyltransferase and down-regulation of fatty acid biosynthesis genes indicate that bile stress changes the flux of acetyl-CoA from fatty acid synthesis towards generation of ATP.

Three transporter and binding proteins were identified (EF0020, EF0709 and EF0912). The IIAB component of a mannose-specific phosphotransferase system (PTS; EF0020) was strongly up-regulated. Interestingly, there are studies showing that this type of transporter is important for the survival of saprophytic and pathogenic bacteria on the mucosal surfaces of animals [35]. Thus, it is conceivable that these proteins are also important for adapting to bile-rich environments. Both the phosphocarrier protein HPr (EF0709) and the peptide ABC transporter (EF0912) were down regulated. The phosphocarrier protein HPr is a component of the phosphoenolpyruvate-dependendent PTS, which is a major carbohydrate transport system in bacteria. The mechanism involves the transfer of a phosphoryl group from phosphoenolpyruvate which are formed by the actions of enolase (EF1961) in the glycolysis. The fact that both enolase (EF1961), and phophocarrier protein HPr (EF0709) are down- regulated, correlates with the down-regulation of other proteins involved in carbohydrate metabolism (see above). In bifidobacteria, bile stress resulted in the up-regulation of some sugar transport proteins [23,28]. This may indicate that *E. faecalis *V583 and bifidobacteria use different strategies in how to handle bile.

Four of the identified proteins have unknown functions, and another ten are hypothetical. Most of these fourteen "unknown" proteins were down-regulated by bile. Only two of these proteins (EF3184, EF3186) were also found to be down-regulated in the transcriptome study [15]. A Blast search with the hypothetical proteins did not reveal significant sequence similarity with hypothetical proteins found in studies of bile responses in other Gram positive bacteria. It must be emphasized that several of these hypothetical proteins are strongly regulated, indicating that they may play important roles in the bile stress response.

Two of the proteins that were classified as proteins with unknown function according to JCVI, are a DNA binding response regulator (EF1193) that is part a two-component regulatory system (EF1194 is a kinase), and an oxidoreductase (EF1138). Two-component regulatory systems are important for responding to environmental changes, whereas the oxidoreductase could play a role in bile modification [13].

Bile salt hydrolases (BSH) catalyze the deconjugation of bile salts, which may be a detoxification mechanism [36]. Experimental data suggests that BSHs indeed play a role in bile tolerance in Gram-positive bacteria [37-39]. The genome of *E. faecalis *contains two genes that putatively encode for BSHs. However, in response to bile no effect on the BSH expression was observed, neither at the transcriptome [15] nor at the proteome level, which is in agreement with other studies on bile-induced gene regulation in Gram-positive bacteria [13,27]. Several gene-by-gene studies indicate that at least part of the known (putative) *bsh*-genes play minor roles in the bile response of lactobacilli [40-42]. All in all, the role of BSHs in bile responses remains somewhat elusive.

## Conclusions

The present study showed that of approximately 500 observed proteins, 53 proteins were significantly regulated in response to bile which provides several leads for further analyses of how *E. faecalis *responds to bile and, perhaps, to stress in general. The clear bile-induced regulation of fatty acid biosynthesis shown at both the proteome and transcriptome level in *E. faecalis *V583 has not been shown previously in Gram-positive bacteria, including enterococci, bifidobacteria and lactobacilli. Proteins involved in fatty acid metabolism were overrepresented among the regulated proteins. In addition, several hypothetical proteins also stand out as targets for further work, including EF1967 and EF2104, which are both abundant and clearly regulated. Such further work is currently in progress in our laboratories.

## Competing interests

The authors declare that they have no competing interests.

## Authors' contributions

IFN, VGHE and GM developed the initial concept for this study. LAB, EMF, IFN, VGHE and GM participated in experimental design. LAB carried out the 2D electrophoresis and did the mass spectrometric analyses. EVK and HS participated in the set-up and standardization of the 2D-electrophoresis. LAB and EMF did the statistical analyses and the initial interpretation of the results. LAB, VGHE and GM drafted the paper, implementing contributions from all other authors. All authors have read, corrected and approved the final manuscript.

## Supplementary Material

Additional file 1 **Figure S1**. Silver stained 2D-electrophoresis gels of the intracellular proteome of *E. faecalis *V583 grown in liquid BHI with and without 1% bovine bile. The gels show protein extracts from cells harvested 20, 60 or 120 minutes after the addition of bile. The numbered spots indicate proteins that were identified as being regulated in response to bile stress, using statistical methods and cut-off values described in the main manuscript. Click here for file
