# Supplementary material for: Identification of proteins related to the stress response in Enterococcus faecalis V583 caused by bovine bile
Source: Proteome Sci. 2010 Jun 25;8:37. doi: 10.1186/1477-5956-8-37 (PMC2907315; doi:10.1186/1477-5956-8-37)
Supplement: Additional file 1 — Figure S1. Silver stained 2D-electrophoresis gels of the intracellular proteome of E. faecalis V583 grown in liquid BHI with and without 1% bovine bile. The gels show protein extracts from cells harvested 20, 60 or 120 minutes after the addition of bile. The numbered spots indicate proteins that were identified as being regulated in response to bile stress, using statistical methods and cut-off values described in the main manuscript. [file 1477-5956-8-37-S1.PDF]

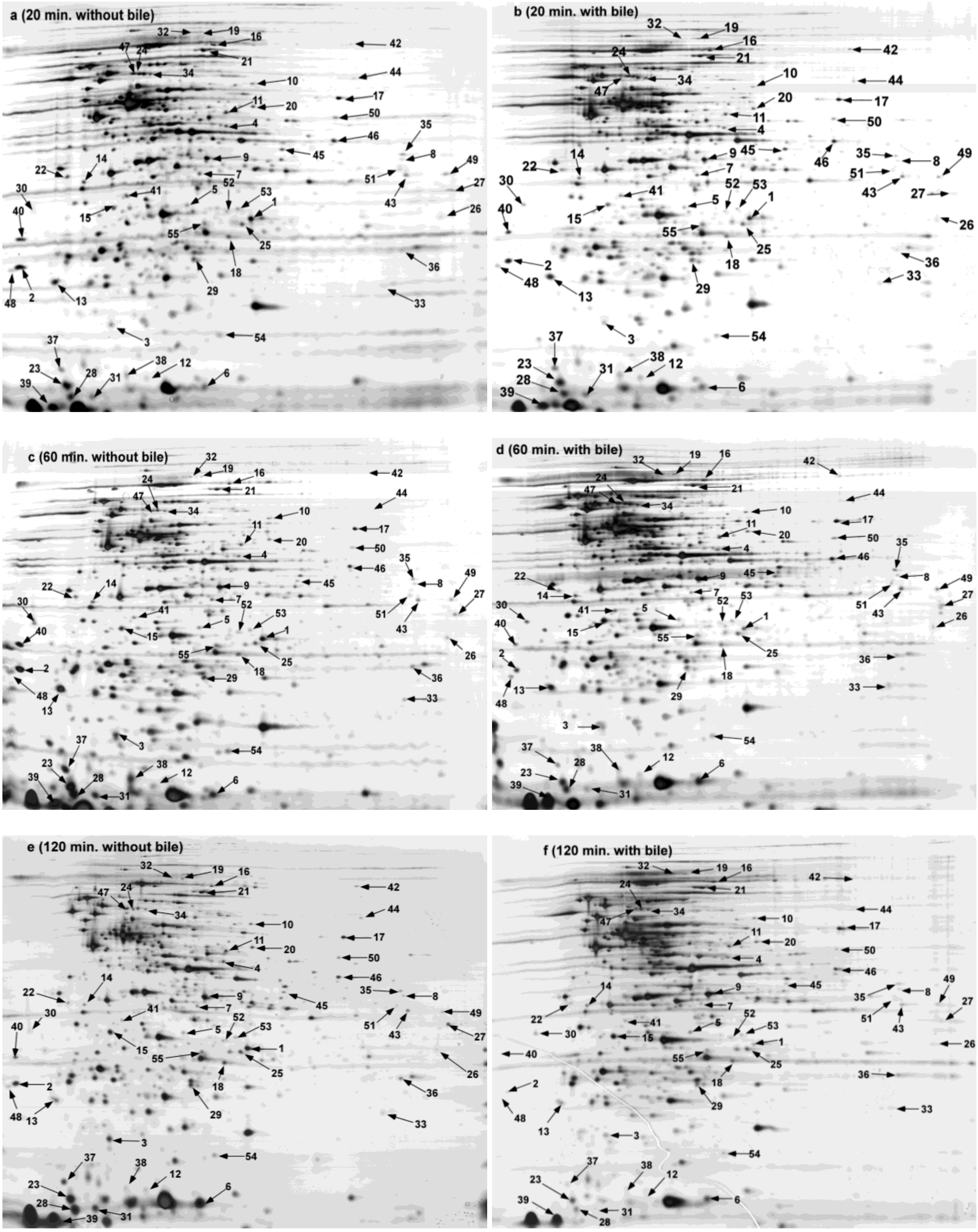

Additional file 1. Figure S1. Silver stained 2D-electrophoresis gels of the intracellular proteome of *E. faecalis* V583 grown in liquid BHI with and without 1% bovine bile. The gels show protein extracts from cells harvested 20, 60 or 120 minutes after the addition of bile.The numbered spots indicate proteins that were identified as being regulated in response to bile stress, using statistical methods and cut-off values described in the main manuscript.
